# Growth Factors Delivery System for Skin Regeneration: An Advanced Wound Dressing

**DOI:** 10.3390/pharmaceutics12020120

**Published:** 2020-02-03

**Authors:** Marta Nardini, Sara Perteghella, Luca Mastracci, Federica Grillo, Giorgio Marrubini, Elia Bari, Matteo Formica, Chiara Gentili, Ranieri Cancedda, Maria Luisa Torre, Maddalena Mastrogiacomo

**Affiliations:** 1 Department of Internal Medicine (DIMI), University of Genova, viale Benedetto XV 10, 16132 Genova, Italy; nardinimarta88@gmail.com; 2 Biotherapy Unit, Ospedale Policlinico San Martino IRCCS, largo Rosanna Benzi 10, 16132 Genova, Italy; 3 Department of Drug Sciences, University of Pavia, viale Taramelli 12, 27100 Pavia, Italy; sara.perteghella@unipv.it (S.P.); giorgio.marrubini@unipv.it (G.M.); elia.bari@unipv.it (E.B.); marina.torre@unipv.it (M.L.T.); 4 Ospedale Policlinico San Martino IRCCS, largo Rosanna Benzi 10, 16132 Genova, Italy; luca.mastracci@unige.it (L.M.); federica.grillo@unige.it (F.G.); matteo.formica@unige.it (M.F.); 5 Anatomic Pathology, Department of Integrated Surgical and Diagnostic Sciences (DISC), University of Genova, viale Benedetto XV 6, 16132 Genova, Italy; 6 Orthopedic Clinic, Department of Integrated Surgical and Diagnostic Sciences (DISC), University of Genova viale Benedetto XV 6, 16132 Genova, Italy; 7 Regenerative Medicine Laboratory, Department of Experimental Medicine (DIMES), University of Genova, via Leon Battista Alberti 2, 16132 Genova, Italy; chiara.gentili@unige.it; 8 Center for Biomedical Research (CEBR), University of Genova, viale Benedetto XV 9, 16132 Genova, Italy; 9 Endolife S.r.l., Piazza della Vittoria 15/23, 16121 Genova, Italy; ranieri.cancedda@unige.it

**Keywords:** advanced wound dressing, human platelet lysate, sericin, alginate, wound healing, regenerative medicine

## Abstract

Standard treatments of chronic skin ulcers based on the direct application of dressings still present several limits with regard to a complete tissue regeneration. Innovative strategies in tissue engineering offer materials that can tune cell behavior and promote growth tissue favoring cell recruitment in the early stages of wound healing. A combination of Alginate (Alg), Sericin (SS) with Platelet Lysate (PL), as a freeze-dried sponge, is proposed to generate a bioactive wound dressing to care skin lesions. Biomembranes at different composition were tested for the release of platelet growth factors, cytotoxicity, protective effects against oxidative stress and cell proliferation induction. The highest level of the growth factors release occurred within 48 h, an optimized time to burst a healing process in vivo; the presence of SS differently modulated the release of the factors by interaction with the proteins composing the biomembranes. Any cytotoxicity was registered, whereas a capability to protect cells against oxidative stress and induce proliferation was observed when PL was included in the biomembrane. In a mouse skin lesion model, the biomembranes with PL promoted the healing process, inducing an accelerated and more pronounced burst of inflammation, formation of granulation tissue and new collagen deposition, leading to a more rapid skin regeneration.

## 1. Introduction

The care of chronic skin ulcers still remains an important problem to be resolved. Skin wound healing requires the recruitment and activity of different cell types such as native immune response cells, endothelial progenitors, keratinocytes, and fibroblasts. The therapeutic approach to a chronic ulcer treatment should take in consideration the morbidity state of the patient (e.g., vascular diseases or diabetes mellitus) and its age, especially when advanced, in order to choose the best strategy. Indeed, the ideal wound dressing should have different properties to consider the different wound requirements.

A lot of different wound dressings have been developed and classified on their action: inert/passive, interactive, and bioactive. The inert/passive ones are ordinary dressings, that only act as covering for the protection of the wound [1]. The dressings capable to influence the regeneration of the damaged surface by modifying the environment are defined interactive. This type of dressing can support all stages of wound healing such as debridement, granulation tissue formation and re-epithelialization and also can reduce the exudate formation and bacterial colonization. Well-known interactive dressings are alginate, collagen and hyaluronic acid-based products [1]. Despite their efficacies, these dressings cannot solve deeper lesions, so bioactive dressings have been developed able to release active substances, like antimicrobials and antibiotics. These dressings maintain the characteristics of the interactive ones with the advantage of the encapsulated hydrophilic and hydrophobic molecules to ameliorate the healing process thanks to their gradual release by the bioactive dressings. However, all these dressings present some limitations with regard to the complete skin ulcer healing and skin damage repair. In spite of the fact that we are still waiting for the “ideal” dressing, marketing studies estimated the arising costs for global wound dressing business in next 5 years by 5% [2], and this, together with the need of better dressings to solve the unresolved clinical problems, is part of the wide interest in the development of new matrices and scaffolds to regenerate the cutaneous tissue.

In this scenario, platelet-derived products, such as platelet gel, have been used in the clinical practice for various reparative treatments since 1986 [3] with the aim to promote or facilitate the wound healing process. In literature the effect of platelet concentrate in the regeneration of soft tissue in term of wound healing, inducing pain relief and subsequently improving the life quality of the patient [4,5] associated with a reduction of hospital stay and of the related costs has been widely discussed. Several Clinical Trials using platelet derivatives have reported interesting results but sometimes controversial. The limits of this approach were attributed to the lack of standardized procedures in the platelet derivatives protocols and interindividual variability of blood donation. Part of this has found an answer by its characterization in terms of platelet concentration and contained growth factors [6,7].

Platelet Rich Plasma (PRP) gel is a matrix derived from the activation of a platelet concentrate in the presence of cryoprecipitate (plasma fibrinogen, fibronectin, clotting factors), thrombin and calcium gluconate. Platelets activation leads to the polymerization of fibrinogen in fibrin forming a 3-dimensional mesh with entrapped platelets that release their growth factors content. This occurs in a wound bed, where the platelets adhere to the sub-endothelium exposed collagen forming a platelet clot of fibrin [8]. The platelet growth factors recruit mesenchymal, epithelial and endothelial cells into the site of the lesion, inducing them to deposit collagen, glycosaminoglycans and other extracellular matrix molecules. The synergic effect among different factors contained in different concentration, as PDGF, VEGF, VFGF, TGFβ, etc. and the temporality with which the factors are released determine the correct wound healing process. Only the regular space-time release of the factors will determine the normal wound healing process. In this context, the platelets coordinate physiologically the release of their growth factors, a mixture of regenerative stimuli acting to promote the natural wound healing process.

In this direction, various typology of scaffolds [9,10,11] were proposed to construct medical dressings for treatment of skin ulcers with the goal to produce easy-to-use medical devices. More recently, in a wide range of biomaterials with the main property of sustaining cell proliferation for new tissue formation and maturation, the silk proteins (fibroin and sericin) were proposed for their good biocompatibility, optimal mechanical properties, and controlled biodegradability profile to obtain bioactive scaffolds for wound healing applications [12,13,14]. In particular, Silk Sericin (SS) presented some biological properties that could support the healing process: ROS-scavenging [12,15], immunomodulatory [13,16], moisturizing [17], anti-tyrosinase and anti-elastase activities [12]. All this evidence encouraged us to select SS as bioactive compound to produce sponge-like dressings for wound healing.

In addition to silk proteins, other polymeric biomaterials were proposed for regenerative medicine purposes, such as alginate, chitosan and hyaluronic acid [1]. Alg is a versatile natural polymer composed by β-(1-4) linked d-mannuronic acid and β-(1-4) linked l-guluronic acid units, able to form hydrogel in presence of calcium ions, typically present in wound exudates. Furthermore, was well demonstrated that alginate is biocompatible, not toxic and able to reduce bacterial infections [18]. For all these reasons, alginate-based dressings and auto-gelling devices [19] were studied and, in some cases, commercialized [20].

Considering the optimal mechanical properties of SS, Alg and the regenerative capability of the PL, we propose their combination to generate an advanced wound dressing for the treatment of chronic skin defects. The binding of PL to SS allows a controlled release of several different growth factors on the site of the lesion in time, while the Alg represents a microenvironment conducive to regenerative stimuli. Given the ability of the PL to induce a burst of inflammation—initial step of the regenerative process—and a good level of cell proliferation, we evaluated the biological effect in vitro and in vivo of a scaffold strongly enriched with growth factors in inducing wound healing.

## 2. Materials and Methods 

### 2.1. Biomembranes Preparation

Low viscosity sodium alginate (Alg) was purchased by Sigma-Aldrich (St. Louis, MO, USA). Platelet derivative (PL), a mixture of platelet growth factors, was derived from PRP preparations with a standardized platelet concentration and produced in a lyophilized form (Lyset ™ CSI087320, Sclavo Diagnostics International, Siena, Italy).

Bombyx mori cocoons (Orgosolo strain) were degummed in autoclave (Systec V-65, Wettenberg, Germany) at 120 °C for 1 h [12]. Obtained Silk Sericin (SS) water solution was dried using a Mini Spray Dryer B-290 (Büchi, Cornaredo, MI, Italy). The SS obtained powder was stored at −18° C until use.

Three different formulations were developed for the study: a biomembrane composed of Sericin and Alginate as negative control and two biomembranes additionally supplemented with PL, as treatment groups. Alg, SS and PL were solubilized in distilled water under magnetic stirring obtaining three solutions (Table 1). 500µL of solution containing mixed components (Alg, SS or PL) were distributed to each well of a 24 well-plate and were freeze-dried (Modulyo^®^ Edwards Freeze dryer, Kingston, NY, USA). The sponge-like dressings were maintained at −20 °C until use.

### 2.2. Fourier Transform Infrared (FTIR) Spectroscopy

Fourier transform infrared spectroscopy was performed on both biomembranes and single components (SS, PL, and Alg). Samples were analyzed using a Spectrum One Perkin-Elmer spectrophotometer (Perkin Elmer, Wellesley, MA, USA) equipped with a MIRacle^TM^ ATR device (Pike Technologies, Madison, WI, USA). The IR spectra in transmittance mode were obtained in the spectral region of 4000–650 cm^−1^, with a resolution of 4 cm^−1^. Measurements were carried out at least in triplicate.

### 2.3. In Vitro Analysis

#### 2.3.1. Growth Factors Release 

In vitro release tests were performed in transwell dishes: 300 µL of physiological solution were placed on the well bottom and 100 µL inside the transwell where the biomembrane was placed. To allow the protein content release through the transwell, incubation was performed at 37 °C and 5% CO_2_. The solutions containing the released proteins were harvested from the well bottom at 30 min, 2, 4, 24, 48, 96, 120, and 144 h. After the recovery, the same starting volume of fresh solution was replaced. Approximately 350 µL of conditioned medium was recovered at each time point. The supernatants were aliquoted and stored at −20 °C for further analysis. For all the analysis three type of biomembranes were tested (Table 1). Alg/PL and Alg/SS/PL biomembranes were used for the evaluation of growth factors release. Alg/SS biomembrane was used as a control to evaluate if SS released from biomembrane lead to a background interference in the evaluation of proteins and growth factors release.

The total protein content in the recovered samples was determined with Pierce™ BCA Protein Assay Kit (ThermoFisher Scientific, Waltham, MA, USA). Vascular Endothelial Growth Factor (VEGF), Platelet-derived Growth Factor-BB (PDGF-BB) and Transforming Growth Factor Beta 1 (TGF-β1) were evaluated by ELISA assays (Human PDGF-BB ELISA kit, RayBiotech Inc, Peachtree Corners, GA, USA; Human VEGF ELISA kit, Invitrogen, Carlsbad, CA, USA; Human TGF-β1 Duo set ELISA R&D Systems, Minneapolis, MN, USA) according to the manufacturer’s instructions. All measurements were performed in duplicate on three independent experiments. By these analyses, we determined the time and the percentage of growth factors released respect to the amount of platelet lysate loaded on each biomembrane.

#### 2.3.2. Cell Cultures

All cell cultures used in this work were obtained from discarded samples of patients undergoing different surgical interventions. All patients previously signed an informed consent approved by the Ethical Committee of San Martino Hospital of Genova, Italy (approval n° MR-10.001, 11.22.2010).

(A) Human bone marrow mesenchymal stromal cells (BMSC) were obtained from femoral heads of patients undergoing surgery for hip rupture. The bone marrow was washed 5 times with Phosphate Buffered Saline (PBS). The cell suspension was centrifuged at 1500 rpm for 10 min, the supernatant discarded, and the pellet re-suspended in the appropriate volume of α-MEM (Lonza, Belgium) supplemented with 100 UI/mL penicillin, 100 μg/mL streptomycin and 2 mM L-glutamine (EuroClone S.p.A, Milan, Italy) Serum-Free Medium (SFM) for the counting of the cells. Counting was carried out using a nuclear dye (0.1% methyl violet in 0.1M citric acid). Cells were plated at a density of 5 × 10^6^ nucleated cells/10 cm Ø Petri dish and allowed to adhere. The cells were cultured in α-MEM supplemented with 10% Fetal Bovine Serum (FBS-ThermoFisher Scientific, Waltham, MA, USA), 100 UI/mL penicillin, 100 μg/mL streptomycin and 2 mM l-glutamine (FBS condition). At confluence, the cells were detached using 0.05% trypsin-0.01% EDTA (EuroClone S.p.A, Milan, Italy), and re-plated at a density of 25 × 10^4^ cells/10 cm Ø Petri dish for cellular expansion.

(B) Human skin fibroblasts (h-FB) were obtained from discarded human skin fragments from patients undergoing reconstructive mastoplasty surgery.

The skin was separated from the fat layer and the extracted pieces disinfected by immersion in 70% ethanol followed by two washes in sterile 1X PBS. The superficial skin was then crumbled using scalpels into ~1 mm pieces that were transferred to 10 cm Ø Petri dish. A sterile slide was put over the skin fragments to favor the isolation of cells from the tissue. Culture was performed in FBS condition. Fibroblasts started to exit from the fragments within 7 days. At confluence, fibroblasts were detached using 0.05% trypsin-0.01% EDTA, and re-plated at a density of 25 × 10^4^ cells/10 cm Ø Petri dish for cellular expansion.

#### 2.3.3. Cell Viability Assay

The toxicity of the components contained in the biomembrane was evaluated by Thiazolyl blue staining (MTT Sigma-Aldrich, St. Louis, MO, USA) on BMSC and hFB cultured in SFM supplemented with SS (1%), PL (5%) and PL:SS (5% and 1% respectively) and in SFM as control. BMSC and hFB at a density of 1 × 10^3^ cells per well were plated in a 96-well plate in FBS condition. After 24 h, the cells were washed with PBS to remove the FBS and exposed to the different treatments. Cell staining was performed at 0, 24, 48 and 72 h after treatment. At each time point, cells were incubated with MTT for 3 h, then the MTT solution was removed and 100 μL of absolute ethanol was added per well to solubilize the formazan product. The reduction of MTT to formazan was quantified by reading the absorbance at 570 nm and 670 nm. This analysis was not performed on Alg because it has been already reported in literature its biocompatibility [21]. 

#### 2.3.4. SS and PL Protective Effect against Oxidative Stress

BMSC and hFB were seeded as described above and treated in the same way. Oxidative stress was induced by treating the cells with 1mM H_2_O_2_. The H_2_O_2_ was added at each culture condition (SS (1%), PL (5%) or PL:SS, 5% and 1% respectively) and maintained for 24, 48 and 72 h. The antioxidant capacity of SS was evaluated by the MTT assay described above.

#### 2.3.5. BrdU Assay

To synchronize the cells before to start the treatments, BMSC were placed in SFM for 24, 48 and 72 h [22]. To evaluate the residual proliferation of BMSC, a Bromodeoxyuridine (BrdU) assay (Roche, Basel, Switzerland) was performed according to the manufacturer’s instructions. Briefly, at the different times, we labeled the cells with BrdU and after 24 h we added Anti BrdU POD for additionally 90 min. We then washed the cells to remove the excess of Anti BrdU POD, performed the substrate reaction and measured the sample absorbance in an ELISA plate reader at 370 nm. After 72 h in SFM the cells did not show any proliferation and we considered these cells synchronized. 

To evaluate the capability of the different treatments to induce proliferation of the synchronized cells, SFM supplemented with 1% SS or 5% PL or PL:SS 5% and 1% respectively was added and a BrdU assay performed at different times (4, 8, 24 and 48 h) after treatment.

#### 2.3.6. Western Blotting

The Western Blot analysis (WB) was performed on BMSC plated in 6 cm Ø Petri dish at a density of 1 × 10^4^ cells. After 72h in SFM, to the cells was added fresh medium supplemented with 1% SS or 5% PL or 1% PL:SS 5% and 1% respectively at different time points (8 and 24 h). To perform the cells lysis the Petri dishes were transferred on ice. The medium was removed, and the cells washed with cold 1X PBS. A lysis buffer (100 μL per 6 cm Ø Petri dish) was added to each plate. To avoid protein degradation, phosphatases and proteases inhibitors (PhosStop™ and cOmplete™, Roche, Basel, Switzerland) were added together with the lysis buffer. The lysis buffer was allowed to act for 5 min, keeping the plate on ice. Then, the cells were dislodged from the plate and transferred to a 1.5 mL tube. The tube was placed on ice for 40 min and vortexed every 10 min. The samples were centrifuged at 4 °C for 20 min at 13,000 rpm, the cell debris pellet discarded and the supernatant containing the proteins collected and stored at −20 °C for further protein analysis. The protein content was quantified by a Bradford protein assay (SERVA, Heidelberg, Germany) according to the manufacturer’s instructions. 

The WB was used to evaluate the expression of Cyclin D1 of the synchronized cells stimulated by the different treatments. Electrophoresis was performed in reducing conditions using 40 μg of protein loaded on a NuPAGE™ 4–12% Bis-Tris gel (Invitrogen, Carlsbad, CA, USA). Proteins were transferred to Amersham™ Protran^®^ 0.45 µm NC nitrocellulose blotting membrane (Sigma-Aldrich, St. Louis, MO, USA). The blot was saturated with 5% skimmed cow milk in TTBS (20 mM Tris HCl pH 7.5, 500 mM NaCl, 0.1% Tween 20) for 2 h at room temperature, washed several times with TTBS and probed in a cold room overnight with specific primary antibodies against Cyclin D1 (1:10,000 Abcam, Cambridge, UK) and β-Tubulin (1:2000, Sigma-Aldrich, St. Louis, MO, USA). After the incubation with the primary antibody, the membrane was washed with TTBS at room temperature for 1 h and incubated with 1:5000 Mouse/Rabbit specific HRP-conjugated secondary antibody (GE Healthcare, Chicago, IL, USA). The specific protein bands of WB were detected with enhanced chemiluminescent (ECL Western Blot Detection Reagent, GE Healthcare, Chicago, IL, USA) and exposure to an X-ray film (GE Healthcare, Chicago, IL, USA). Densitometric analysis of the film was performed using the Epson Perfection V330 Photo scanner (Epson, Milan, Italy) and the band densities were quantified with ImageJ software version 5.2.5 (5.2.5) (https://imagej.nih.gov/ij/download.html).

### 2.4. In Vivo Mouse Wound Healing Model

All experimental animal procedures were evaluated and approved by Ethics Committee for animal experimentation (CSEA) and communicated to the Italian Ministry of Health in accordance with article 31 of the D.lgs 26/2014 (approval n° 787, 10/16/2017 by Italian Ministry of Health).

In vivo tests were performed to determine the efficacy of the biomembranes [23]. 6 weeks C57/BL6 wt mice were anesthetized with xylazine/ketamine and depilated on the dorsal surface with a depilatory cream. On the depilated dorsal surface, two full-thickness wounds were made with a circular 6 mm punch for each animal. A sponge loaded with PL (treated) or an unloaded sponge (control) was applied over the wound and covered with a transparent patch (Tegaderm, 3M, Milan, Italy) to protect the wound from contacts with external agents. At the end of the surgical procedure, mice were returned to their cages and kept under red light to compensate for the hypothermic effect of the anesthesia. Every two days the wounds were photographed. The mice were euthanized with carbon dioxide at 2, 3, 7, 14, and 21 days after surgery and the skin lesion area collected and processed for histology. The groups of animals sacrificed at 3, 7 and 14 days were composed of six animals; and at 2 and 21 days three animals per group.

### 2.5. Histology

Skin samples were fixed with 10% buffered formalin at room temperature for 24 h, routinely processed and paraffin-embedded for tissue sectioning. 3 μm thickness sections were prepared and stained with hematoxylin-eosin and Masson’s trichrome. The evaluation of inflammation, granulation tissue, and neovascularization was performed following guidelines provided by Abramov et al. [24] which quantify different parameters giving them a score from 0 to 3. Briefly, early (acute) inflammation, late (chronic) inflammation, granulation tissue amount and collagen deposition were scored as: 0-none, 1-scant, 2-moderate, 3-abundant; fibroblast maturation was scored as: 0-immature, 1-mild maturation, 2-moderate maturation, 3-fully matured; re-epithelization was scored as: 0-none, 1-partial, 2-complete but immature or thin, 3-complete or mature; neovascularization in terms of vessels number: 0-none, 1-up to 5 vessels per high-power field (HPF), 2-6 to 10 vessels per HPF, 3-more than 10 vessels for HPF.

### 2.6. Statistical Analysis

All in vitro experiments were conducted in triplicate on three different batches of biomembranes and using three different primary cell cultures. Statistical analyses were performed using the two-way ANOVA provided by the GraphPad Software version 6.0c (www.graphpad.com). In vivo experiments to test efficacy of Alg/SS/PL biomembranes were repeated six times for times 3, 7 and 14 days and in triplicate for 2 and 21 days. To evaluate differences between treated and control lesions statistical analyses were carried out using Mann–Whitney test.

## 3. Results

### 3.1. FTIR Analyses of Single Components and Biomembranes

**Figure 1:**
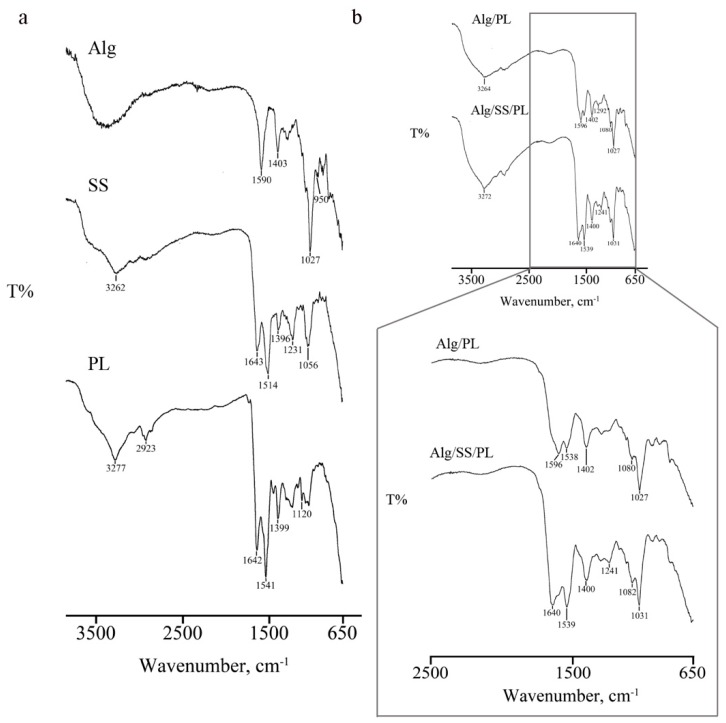
Fourier transform infrared (FTIR) spectroscopy. The analysis was performed on single components (SS, PL and Alg) (**a**) and biomembranes (**b**) The IR spectra where obtained in the spectral region of 4000–650 cm^−1^.

Biomembranes generated by the combination of Alg/PL and Alg/SS/PL in defined ratios were analyzed by FTIR to measure the spectrum of the biomembrane single components. FTIR spectrum of SS (Figure 1a) showed a peak between 1800–1600 cm^−1^ and characteristics of the stretching vibration of C=O groups. Peaks at 3500-3000 cm^−1^ are associated with N–H stretching vibrations. At 1513 cm^−1^ it was possible to show an amide II absorption band; while C=O symmetry stretching was observed at about 1400 cm^−1^ (1397 cm^−1^).

Alg FTIR spectrum (Figure 1a) presented absorption bands around 1590 cm^−1^, 1402 cm^−1^, and 1291 cm^−1^ that are correlated to stretching vibrations of asymmetric and symmetric bands of carboxylate anions. A not well resolute band at about 3400 cm^−1^ is characteristic of –OH stretching vibrations.

FTIR analysis of PL (Figure 1a) showed the characteristics peaks of proteins, as reported by Barth [25]. The NH stretching vibration of amide A and B gives rise two to bands at 3277 and 3061 cm^−1^, respectively. Absorption band at 1641 cm^−1^ is correlated to C=O stretching vibration of amide I, while peaks at 1540 and 1451 cm^−1^ are related to the amide II bending and stretching vibrations (NH and CN groups, respectively). Contributions of amide III-correlated NH bending vibration are visible in the 1400–1200 cm^−1^ region.

After analysis of single components, FTIR was performed on Alg/SS/PL-based biomembrane (Figure 1b) demonstrating the simultaneous presence of absorption bands related to OH stretching vibration of Alg chains and to NH stretching vibration of PL (3400–3000 cm^−1^ region). In the region 1600–1100 cm^−1^ peaks are principally correlated to the amides of PL (1538, 1402, 1121 cm^−1^). An interaction between two components was observed by the shift of absorption bands of Alg, from 1595 to 1590 cm^−1^, and of PL, from 1294 to 1305 cm^−1^. FTIR spectrum of Alg/SS/PL biomembrane was superimposable to the spectrum of control biomembrane (Alg/PL) though a peak at 1639 cm^−1^, attributable to the presence of SS, was well-visible. Moreover, the addition of SS to biomembrane induced the interaction between functional groups of protein chains; in particular, it was possible to observe the presence of a well-defined peak at 1241 cm^−1^, not visible in PL and SS spectra, owing to C–N stretching vibrations in the amide III linkage.

### 3.2. Proteins and Growth Factors Released from the Biomembranes

**Figure 2:**
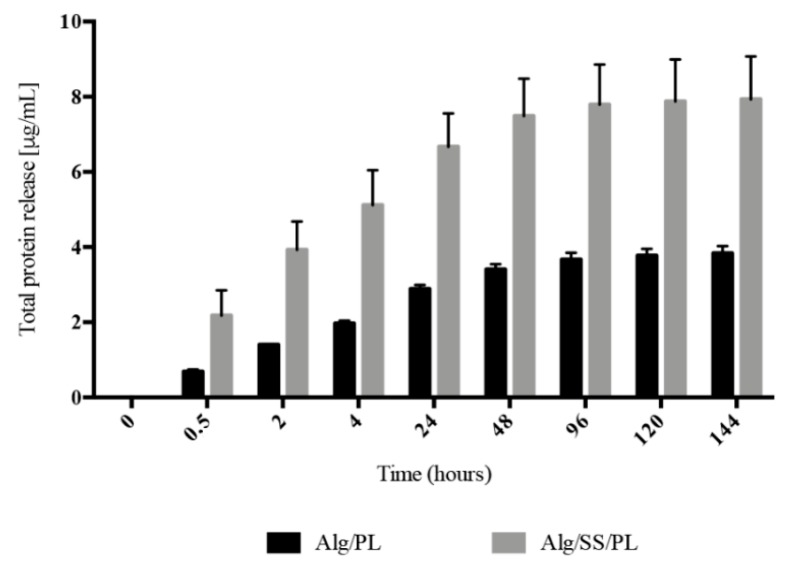
Protein release analysis. Release of total proteins from Alg/PL and Alg/SS/PL biomembranes at different time incubation in physiological solution at 37 °C and 5% CO_2_. The total protein content was determined with Pierce™ BCA Protein Assay Kit (Thermo Fisher Scientific, Waltham, MA, USA) in the incubation media collected at different times. The experiments were performed in triplicate on three different batches of biomembranes.

**Figure 3:**
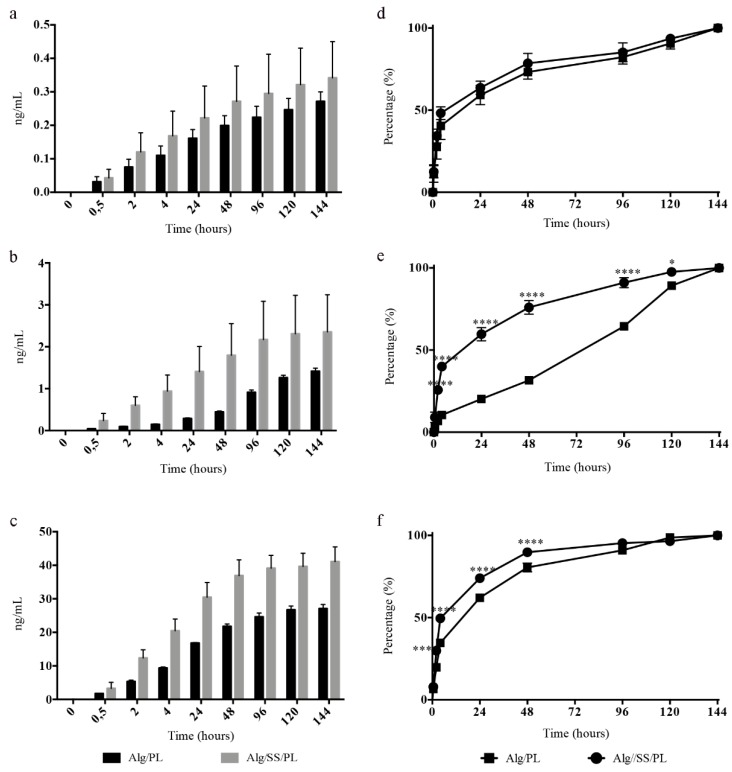
Growth factors release analysis. Release of growth factors from Alg/PL and Alg/SS/PL biomembranes after incubation in physiological solution at 37 °C and 5% CO_2_. The amounts of Vascular Endothelial Growth Factor (VEGF) (**a**), Platelet-derived Growth Factor-BB (PDGF-BB) (**b**) and Transforming Growth Factor Beta 1 (TGF-β1) (**c**) released in the incubation medium at different time points were determined by ELISA assay. The curves represent the release during time of the three different factors expressed as percentage of the maximum release: VEGF (**d**), PDGF-BB (**e**), TGF-β1 (**f**), **** *p* < 0.0001, *** *p* < 0.001, * *p* < 0.05.

The histogram in Figure 2 shows the release of total proteins following the incubation of Alg/PL and Alg/SS/PL biomembranes with physiological solution, at 37 °C and 5% CO_2_ and for different time points. The release from the two biomembranes presented the same trend, but a higher amount of released protein was evident in the incubation medium of Alg/SS/PL biomembrane. This was due to the co-presence of SS protein and PL proteins which are instead present in equal amounts in both types of biomembranes (50% *w*/*w*). By ELISA assay was evaluated the release in the incubation medium of PDGF-BB, VEGF, and TGF-β1 (Figure 3a–c), to monitor the PL growth factors release in time. Interestingly, the presence of SS in the biomembrane determined the release of a higher amount of growth factors. To investigate possible differences in the release of these growth factors in time, the percentage of factors released in the incubation medium was determined from 30 min to 6 days: 100% was considered the growth factor amount released after 6 days, intended as the maximum capability of the sponge to release a specific growth factor (Figure 3d–f). In the presence or absence of SS in the biomembrane, the growth factors analyzed were released more than 50% of the total within the first 24 h. Only for PDGF-BB, in absence of SS, we can observe a delayed release (Figure 3e) without the initial burst of release like registered for the other analyzed factors. For PDGF-BB, a statistically significant difference in the release was observed from 2 to 120 h, while the release trend of TGF-β1 was similar from both types of biomembranes and a statistically significant difference in the percentage of growth factor released was observed only from 2 to 48 h (Figure 3f). No statistically significant differences were observed for VEGF release (Figure 3d).

### 3.3. Biomembrane Biocompatibility

**Figure 4:**
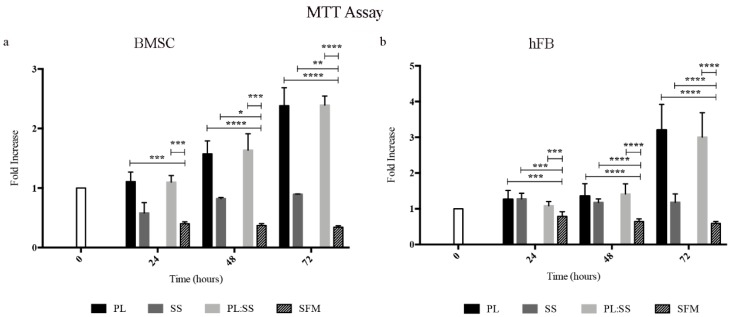
Cell viability with biomembrane components. Analysis of bone marrow mesenchymal stromal cells (BMSC) (**a**) and human skin fibroblasts (hFB) (**b**) viability and capacity of cells to proliferate following the PL stimulation in the presence of SS. Cells were maintained up to 72 h in Serum-free medium (SFM), or SFM supplemented with PL (PL), Sericin (SS), PL and Sericin (PL:SS). Cell viability was determined by a Thiazolyl blue staining (MTT assay), **** *p* < 0.0001, *** *p* < 0.001, ** *p* < 0.01, * *p* < 0.05.

**Figure 5:**
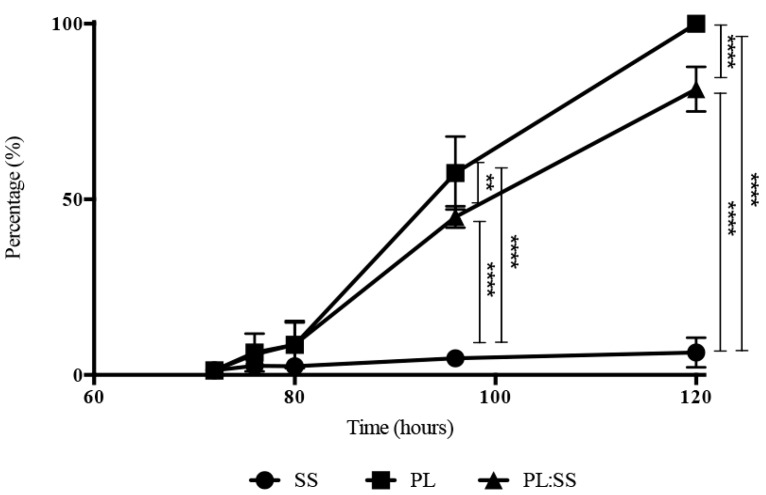
Evaluation of cell proliferation. Proliferation rate of synchronized growth arrested BMSC after stimulation with Sericin (SS), Platelet Lysate (PL), and PL:SS. Data are presented as percentage of the highest growth observed at 48 h in cells stimulated with PL alone. We determined growth rates of three different synchronized primary BMSC cultures in triplicate, **** *p* < 0.0001, ** *p* < 0.01.

Based on the data of the literature demonstrating that Alg is inert and fully biocompatible, the SS and PL were tested for their interference on the viability and proliferation of BMSC and hFB (Figure 4 and Figure 5). In both BMSC (Figure 4a) and hFB (Figure 4b) cultures the switch of the cells from FBS condition to SFM showed a decrease of the cell viability already in the first 24 h remaining unvaried over 72 h. The addition of SS, compared to cultures maintained in SFM, had a beneficiary effect on the vitality of the cells; the cell viability was more supported by PL and SS did not negatively interfere when was associated with PL.

To better evaluate the effect of SS and PL in the cell proliferation, BrdU assay, based on the bromodeoxyuridine incorporation during DNA duplication, was performed on BMSC cultures (Figure 5). The results showed an increased cell proliferation in the presence of PL. SS maintained the cell viability but did not induce proliferation.

**Figure 6:**
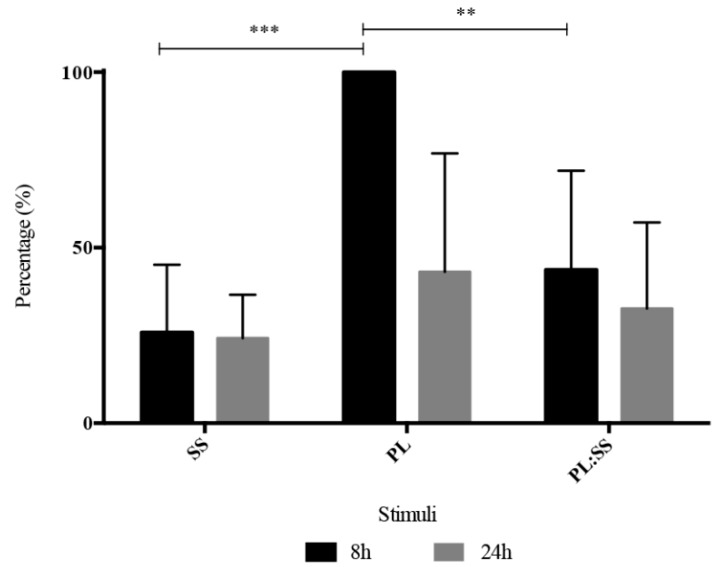
WB analysis of Cyclin D1. Level of Cyclin D1 expression 8 and 24 h after stimulation of synchronized BMSC with SS, PL and with PL:SS. Results are expressed as percentage of the highest expression observed 8 h after stimulation with PL. Levels of Cyclin D1 expression were determined by Western Blot. We performed WB analysis on three different primary cell cultures, *** *p* < 0.001, ** *p* < 0.01.

A corresponding effect was observed evaluating Cyclin D1 expression by Western Blot analysis in synchronized BMSC after 8 and 24 h of PL and SS:PL treatments. The highest stimulatory effect was observed at 8 h for both PL alone and PL:SS (Figure 6).

### 3.4. Protection against Oxidative Stress due to the Biomembrane Components

**Figure 7:**
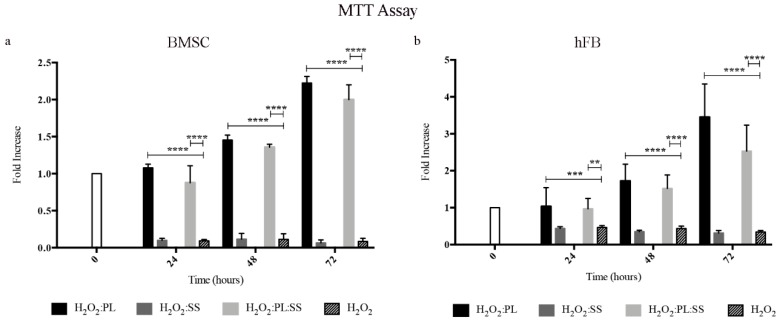
Protection against oxidative stress. Viability of BMSC (**panel a**) and hFB (**panel b**) after an oxidative stress induced by H_2_O_2_ (1 mM) and the effect of a contemporary treatment with Sericin (SS), Platelet Lysate (PL), and PL:SS. Cell viability was determined by MTT assay, **** *p* < 0.0001, *** *p* < 0.001, ** *p* < 0.01.

Considering the documented role of SS in protecting cells against oxidative stress, we performed experiments on BMSC and hFB administering to the cells a dose of hydrogen peroxide (1mM), known to induce an oxidative stress and to be toxic for the cells. The supplement of the culture medium with PL or with PL:SS could rescue the cells from the oxidative stress and induce the cell proliferation, whereas the supplement of SS alone (the amount contained into the biomembrane) did not have any protective effect against oxidative stresses (Figure 7a,b).

### 3.5. Biomembrane Effect on the Healing of a Mouse Excisional Wound Model

**Figure 8:**
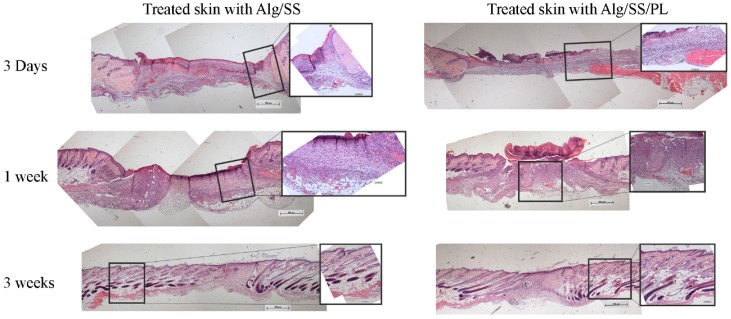
Histological analysis of skin lesions. Histology of lesions treated with Alg/SS and Alg/SS/PL biomembranes. Bioptic sample sections were cut at 3 μm thickness and stained with haematoxylin-eosin. Images were acquired at 5× magnification (0.5 mm scale bar) and 20× magnification (0.1 mm scale bar).

To evaluate the effect of the biomembranes in vivo, we used a mouse excisional wound healing model in C57/BL6 mice. Two full-thickness skin lesions were created in the back of the animals and a biomembrane was applied to each lesion. On each animal, we applied two biomembranes: one without PL as control and one with PL as treatment. We used different combination of sponges: Alg, Alg/SS, Alg/PL and Alg/SS/PL. Evaluation of the biomembrane efficacy was determined by the histological analysis of the samples recovered from the animals at different times from the treatment (2, 3, 7, 14 and 21 days). In Figure 8 we compared the healing of the lesions treated with the Alg/SS/PL and Alg/SS biomembranes. The hematoxylin-eosin staining showed the closure of the lesion after 3 weeks in both the lesions treated with Alg/SS/PL or Alg/SS. However, a more organized tissue similar to a healthy skin was observed in the treated animal group. At 3 days an inflammatory response was evident, which was completely substituted by a granulation tissue at 1 week. The histological analysis included also the evaluation of some specific parameters suggested by Abramov et al. [24]: (1) inflammation, defined in an initial (early) phase by the presence of neutrophils and in a more advanced (late) phase by the presence of plasma-cells and monocytes; (2) granulation tissue, intended as amount and maturation of granulation tissue present. The degree of maturation of the granulation tissue was determined by the shape and the alignment of the fibroblasts; (3) collagen deposition, considering the amount and the degree of maturation of the collagen fibers; (4) re-epithelialization, considering the degree of closure of the lesion; 5) neo-vascularization, considering the number of formed vessels.

**Figure 9:**
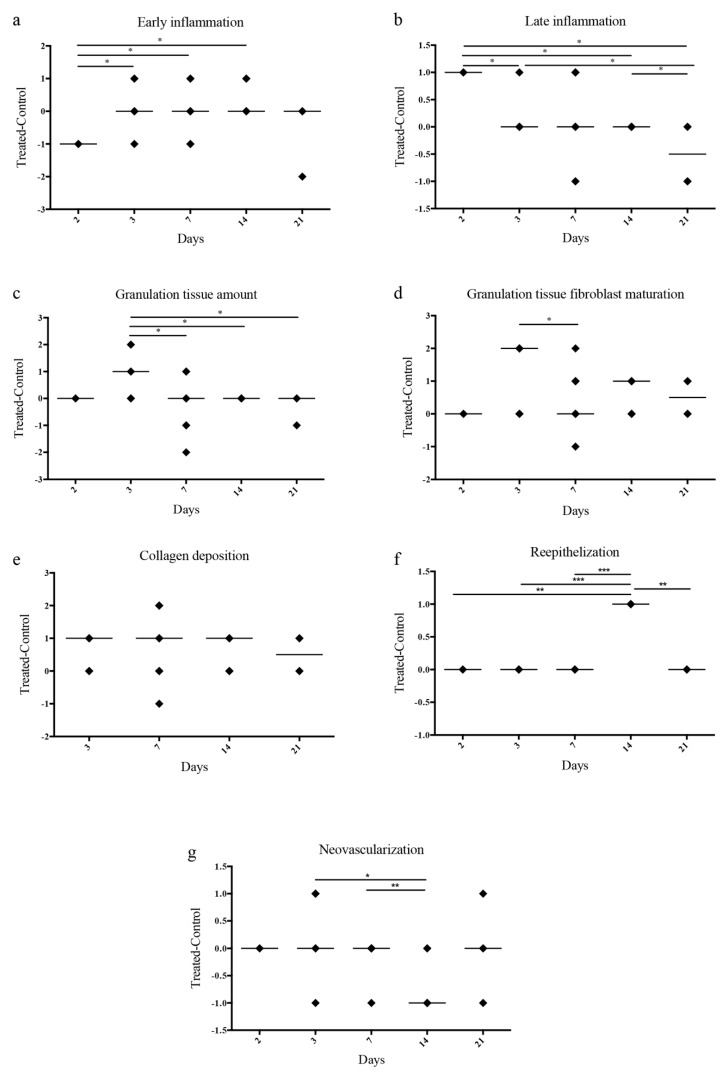
Quantification of histological parameters. Histology of the lesions treated with Alg/SS and Alg/SS/PL biomembranes; evaluation and statistical analyses of specific parameters suggested by Abramov et al. [24]: inflammation, defined in an early phase (**a**) and a late one (**b**); granulation tissue, intended as amount (**c**) and maturation of granulation tissue (**d**); collagen deposition (**e**); re-epithelialization (**f**) and neo-vascularization (**g**). All evaluations were reported in the plot as difference between the PL treated and the control lesions, *** *p* < 0.001, ** *p* < 0.01, * *p* < 0.05.

We analyzed 3 to 6 animals per group. All evaluations were reported in the plot as difference between the PL treated and the control lesions (Figure 9). At day 2 the persistence of an initial inflammatory reaction was observed in the lesions treated with the control biomembrane, whereas a more advanced inflammatory phase was observed in the lesions treated with the PL containing biomembranes. At day 7 there was no statistically significant difference between the control and the PL treated lesions. At day 21 a late inflammation phase was evident only in the control lesions.

The granulation tissue was increased in the PL treated lesions at day 3 whereas no statistically significant differences were observed at all times with regard to the collagen deposition. The re-epithelialization occurred faster in the PL treated wounds. At day 14 an increment in the re-epithelialization of the PL treated lesions was already evident.

No differences in the neo-vascularization of the two lesions groups were observed at days 2, 3, and 7. At day 14 the control lesion presented a more extended network of new vessels. This could be due to the delay in the wound re-epithelialization with a consequent further progression of the granulation phase. In summary, these results were in agreement with an accelerated healing of the PL treated lesions.

## 4. Discussion

In this study, we investigated the combination of two biomaterials, Sericin and Alginate, with a platelet derivative to generate a biomembrane with a controlled growth factors release to be adopted as an advanced medical treatment for skin wound healing process.

Alginate, thanks to its mucoadhesive property, low toxicity and immunogenicity together to the capability to sustain cell activity and to support bioactive molecules release, is commonly used for drug/growth factor delivery.

SS was reported to be beneficial in accelerating cell proliferation and in increasing cell viability of different cell lines and specifically to promote wound healing [26,27]. SS presents many characteristics relevant for biomedical applications, as biocompatibility, low toxicity, mechanical stability, and biodegradability although, in the past, it was only considered a waste product of textile industry. By a degumming process, SS is separated from fibroin with a small contamination (10%) of pigments (flavonoids), sugar, wax, mineral salts and other impurities to which are attributed some antioxidative activities. Based on its properties, SS finds wide consideration in wound healing since encourages collagen formation; it acts as anticarcinogenic, reducing the oxidative stress in cancer cells [28,29], and as anticoagulant, interfering with the fibril gathering of fibrin [30], presents an immunomodulatory activity [12,13] and is a vehicle for drug delivery [31]. The viscosity of its gel allows its use in food and cosmetic [32,33], in contact lens production and in bandage [34]. SS is attracting particular interest in regenerative medicine when used as microspheres carrying molecules with anti-microbial [35] and/or growth factor activity.

In this regard, PL plays important roles during the tissue regeneration by the release of its growth factors. In particular, PDGF stimulates the migration of neutrophils, macrophages to the damage site, participate to the re-epithelization and in angiogenesis processes, but also acts a mitogenic effect for several types of cells. VEGF stimulates the neurogenesis and new blood vessel formation; TGF-β1 has a role in inflammatory process. The presence of these factors and of many others allow one to consider the PL not only a good substitute of FBS for in vitro cell cultures, but in particular a key player in the activation of in vivo regenerative processes. Platelet-derived products, such as PRP, PL, platelet gel, have been used to promote wound healing in the clinical practice for therapy of skin ulcers, osteoarticular regeneration, low back pain management and as antibacterial agent [36,37,38,39]. Considering the controversial literature data [40,41,42,43] on the effectiveness of the PRP, we figured that the inconsistency of the published results was most probably due to a poor quality of PRP produced by inadequate and uncontrolled procedures and to the extreme variability of the PRP product prepared from a single blood donation without considering the significant inter-individual variability in the platelet concentration of human plasma and the variability within the same individual, according to his health status. Therefore, the PL used for the biomembrane production was derived from large pools of blood donations collected according to blood banking safety practices and processed following standardized industrialized procedures [6,7,44]. Our preliminary clinical results have shown that the PRP, prepared according to controlled procedures, has a beneficial effect in the treatment of chronic skin ulcers (data not shown).

On these assumptions, the idea of combining the pro-regenerative effects of PL with natural matrices able to release it on the wound bed led us to design a very manageable biomembrane that we have characterized in vitro and tested in vivo. The amount of protein released by Alg/PL and Alg/SS/PL biomembranes was proportional to the loaded proteins, presented comparable kinetics and in both cases a plateau was reached in 4-6 days. Instead, the release of the PL growth factors was differently modulated depending on the presence or absence of SS in the biomembrane. In particular, VEGF and TGF-β1 were released with a similar trend from the two biomembrane typologies, while the PDGF-BB release occurred differently. About 50% of all three analyzed factors was released within the initial 4 h from the Alg/SS/PL biomembrane. On the contrary, while about 50% of VEGF and TGF-β1 was released within the initial 4 h burst also from the Alg/PL biomembrane, the PDGF-BB release was more progressive and persisted during the 7-day experiment. This behavior could be explained by the presence of SS that enlarged the pores of the Alg matrix. This was confirmed by the FTIR analysis showing that SS interacts with the components, Alg and PL, forming new peaks of absorption bands. This corresponds to a rearrangement of the material structure.

To verify a possible interference with the PL-induced cell proliferation [45,46] by the mixture of the two biomembrane components (SS and PL), we performed cell culture experiments in the presence of different combinations of the components. In our experimental condition, SS did not show any cytotoxic effect and did not interfere with the proliferative effect exerted by the growth factors contained in PL. Further confirmations were obtained by evaluating the cell proliferation by BrdU assay and investigating the expression of cyclin D1. SS alone did not induce in vitro BMSC proliferation while the culture treatment with SS and PL determined a strong proliferative effect similar to the treatment with PL alone, showing that proliferation in the PL:SS supplement was due to the stimuli provided only from growth factors contained in PL. Also cyclin D1, that plays a key role in the regulation of the proliferation, was strongly induced by PL treatment, barely by PL:SS mixture and less after treatment with SS alone. Different results are reported in the literature on human fibroblast cultures [47], where SS used as supplement is able to sustain cell proliferation and the combination with PL induced a higher proliferation rate, suggesting a synergic effect between PL and SS. Further data [48] showed that the mixture SS microparticle/PL induces a high cell viability in cells derived from human nucleus pulposus. The different results we obtained could be due to the synchronization of cell culture system that we performed before evaluating the proliferation.

Considering the described antioxidant properties of SS [48,49] we evaluated if this protein could be responsible of a protective effect against hydrogen peroxide-induced oxidative stress, when conjugated to the PL in the biomembrane. In our experimental conditions, an antioxidant protection role played by SS alone, was not observed while this effect was clearly exerted by the PL presence and cell viability increased until the end of the proliferation analysis (72 h). This effect was demonstrated for both human BMSC and fibroblasts.

These apparently conflicting results with the literature data [48,49] may be attributed to the different protocol of treatment of the cells. In fact, while these authors performed a SS pre-treatment before inducing the oxidative insult, we simulated what occurs within the lesion, and then the contemporaneity of the oxidative stress and the treatment with SS and PL was evaluated.

Following the in vitro characterization of the biomembranes, a comprehensive in vivo study in a mouse skin regeneration model was performed. Biomembranes composed of Alg/SS/PL and Alg/SS were applied on the back of mouse where a critical skin lesion was created. From the histological analysis of the samples, recovered at different time after the application, we observed that Alg/SS/PL biomembrane led to a faster regeneration of the skin wound compared to the control (Alg/SS). The inflammation phase occurred faster in PL treated lesion, rapidly leading to the formation of granulation tissue and the deposition of new collagen, in agreement with several previously reported studies [50]. The same sequence of events was occurring, but at a slower rate in the control lesions treated by the biomembrane without PL. In addition, the re-epithelization phase, that occurred in two weeks in the PL treated lesions, was delayed in the control lesions, possibly because of the persistence of neovascularization. To our knowledge, to date no other data are reported in the literature on the regenerative effect of a SS/PL-based dressing on wound healing in vivo. Our data are supported by histological studies performed on human skin strips that showed the positive effect of PL loading dressings, containing chitosan glutamate, sericin, and glycine, on dermal matrix reconstruction [47]. An interesting point is derived from growth factors release results’ that correlate to the tissue regeneration obtained in vivo, demonstrating that the initial burst of PDGF-BB released from Alg/SS/PL biomembrane facilitates the activation of a cascade of reactions leading to the healing process, respect to Alg/PL biomembrane that showed a gradual release of the same growth factor.

Further demonstration to support the role of SS in tissue regeneration were preliminarily in vivo tested, demonstrated that the biomembrane containing Alg without SS (Alg and Alg/PL) did not show any different effects in the closure of the skin lesion respect to the biomembrane containing SS (data not shown).

In conclusion, this work proposes a manageable freeze-dried wound dressing, composed of Alg and SS in combination with a powerful inductor of cell proliferation, as the PL, for skin lesion treatment. The SS contributes and supports the effect of PL by controlling the PL factor release into the lesion.

## Figures and Tables

**Table 1:** Composition of the biomembranes. Biomembranes percentage composition (*w*/*v*) of solution employed for biomembrane preparation and percentage composition (*w*/*w* %) of freeze-dried biomembranes.

Type of Biomembrane	Name	Solution Percentage Composition (*w*/*v*)			Membrane Percentage Composition (*w*/*w* %)		
		Alg	SS	PL	Alg	SS	PL
Control	Alg/SS	1	1	0	50	50	0
Treated	Alg/SS/PL	1	1	2	25	25	50
Treated	Alg/PL	1	0	1	50	0	50
